# Catheter allotopia with totally implantable access port: A report of three cases and literature review

**DOI:** 10.1002/ccr3.3479

**Published:** 2020-11-06

**Authors:** Jialin Gu, Guoli Wei, Lingchang Li, Yi Ji, Jialin Yu, Canhong Hu, Jiege Huo

**Affiliations:** ^1^ Department of Oncology Affiliated Hospital of Integrated Traditional Chinese and Western Medicine Nanjing University of Chinese Medicine Nanjing China

**Keywords:** catheter allotopia, left internal jugular vein, totally implantable access port, X‐ray radiography

## Abstract

Early detection and treatment are critical for preventing catheter allotopia in the totally implantable access ports and whenever possible, the right internal jugular vein should be selected as the first puncture point.

## Graphical abstract

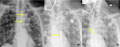

## 1 INTRODUCTION

Catheter allotopia is a common complication associated with totally implantable access ports (TIAPs) that can lead to premature catheter removal or other secondary risks. Timely detection and treatment are critical to achieving a satisfactory outcome.

Totally implantable access ports are safe, efficient, low‐risk, and easy‐to‐maintain medical devices that provide reliable venous access in patients receiving long‐term intravenous infusion. They were first used in cancer therapy in 1982.^1^ There are some not insignificant immediate and long‐term complications associated with TIAPs including catheter allotopia, pneumothorax, arrhythmia, catheter embolism and infection, and pinch‐off syndrome. Catheter allotopia is a common reason for premature catheter removal. Here, we report 3 clinical cases of catheter allotopia in which the TIAP catheter migrated to the azygos vein (AV, Case 1), internal thoracic vein (ITV, Case 2), and lower craniocerebral segment of the right internal jugular vein (IJV, Case 3). We also discuss this issue in the context of the literature.

## 2 CASE REPORTS

### 2.1 Case 1

**FIGURE 1:**
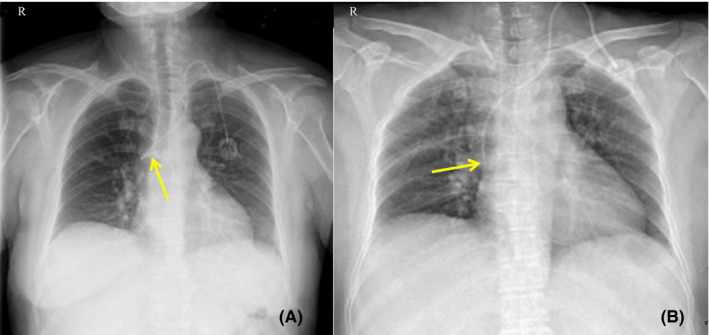
X‐ray radiography findings in Case 1. A, The first postoperative X‐ray radiographic examination showed that the catheter tip had entered AV. B, A second X‐ray examination showed that the catheter tip was correctly repositioned at the level of the 7th thoracic vertebra

A 58‐year‐old woman was diagnosed with ovarian cancer with peritoneal metastasis. We planned to use the docetaxel plus cisplatin regimen for chemotherapy after she underwent ovarian cancer reduction surgery. Since ultrasonic examination revealed that the patient's right IJV was too thin for catheter insertion, left vascular access was selected and the port was subcutaneously implanted in the left infraclavicular fossa. Postoperative X‐ray radiography showed that the catheter had migrated into AV (Figure 1A). We resterilized and reopened the left jugular incision and pulled out the catheter about 5 cm from the puncture point before slowly replacing it. A second X‐ray examination revealed successful repositioning of the catheter (Figure 1B).

### 2.2 Case 2

**FIGURE 2:**
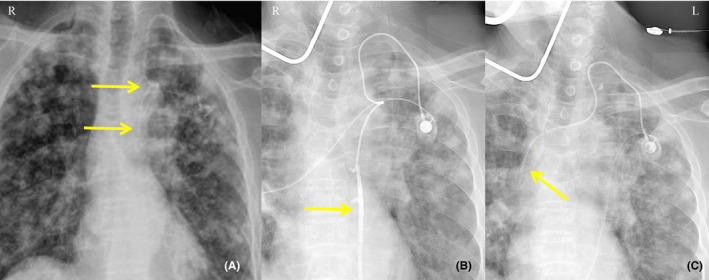
X‐ray radiography and DSA findings in Case 2. A, Postoperative X‐ray radiographic examination revealed abnormal positioning of the catheter tip. B, DSA revealed entry of the catheter tip into the left ITV. C, Correct positioning of the catheter tip at the level of the 6th thoracic vertebra after catheter repositioning as confirmed by DSA

A 74‐year‐old man was diagnosed with stage IV left lung adenocarcinoma. The patient selected TIAP for chemotherapy and intravenous nutritional support. Because of scar tissue hyperplasia at the right puncture point, the left IJV was chosen as the vascular access point. X‐ray radiography revealed abnormal positioning of the catheter after surgery (Figure 2A). Digital subtraction angiography (DSA) showed that the catheter tip had slipped into the left ITV (Figure 2B). We removed the suture and slowly retracted the catheter, which was then reinserted into the superior vena cava when DSA showed that the tip was drawn back into the subclavian vein (SV). X‐ray radiography confirmed the correct positioning of the catheter (Figure 2C).

### 2.3 Case 3

**FIGURE 3:**
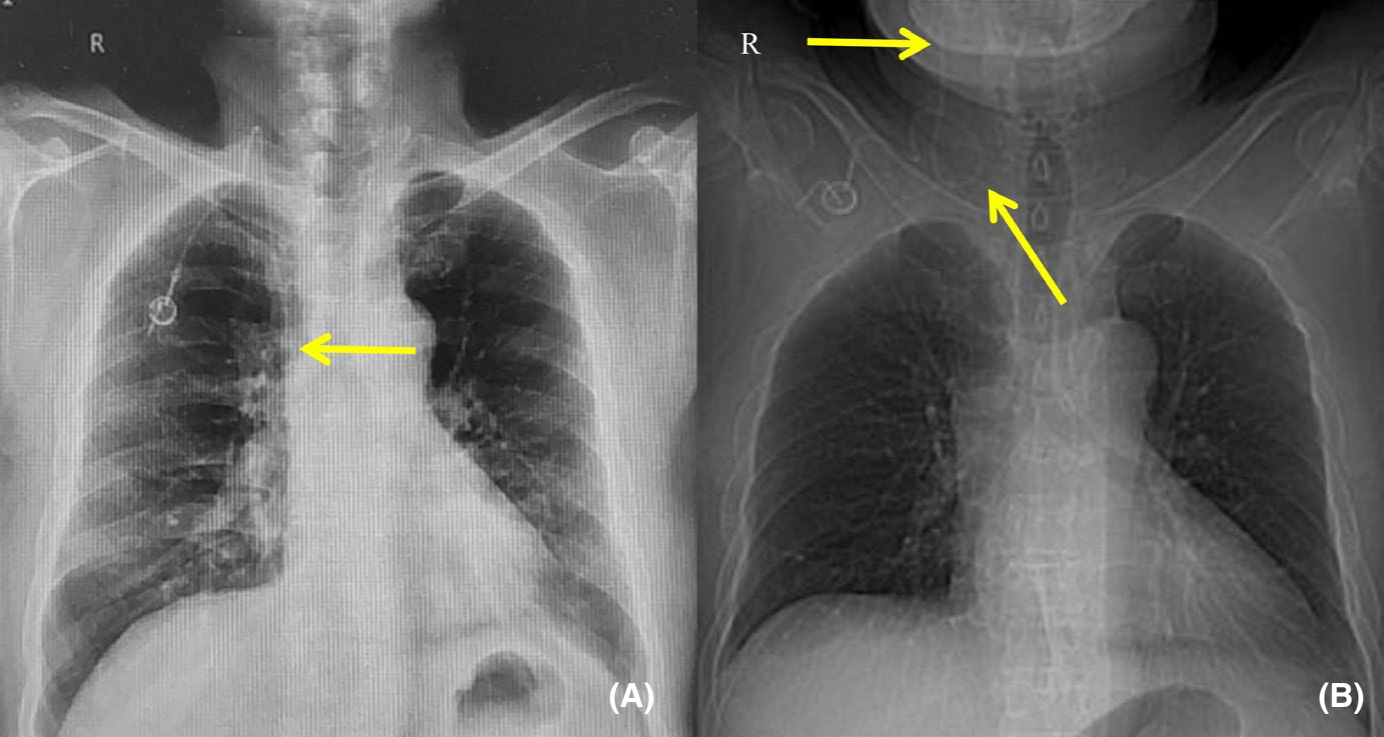
X‐ray radiography findings in Case 3. A, Postoperative X‐ray radiography showed that the catheter tip was correctly positioned. B, A second X‐ray examination revealed that the catheter tip had entered the lower craniocerebral segment of the right IJV

A 63‐year‐old woman was diagnosed with lung adenocarcinoma. The TIAP was implanted on 4 May 2018 by puncturing the right IJV, and the correct positioning was verified by X‐ray radiography (Figure 3A). From May 2018 to July 2019, the patient received molecular targeted therapy and chemotherapy in succession. Since the TIAP can normally be used without any discomfort, no additional X‐ray images were acquired to verify the catheter tip position. On 20 November 2019, the patient was hospitalized for severe headache and fatigue. During transfusion through the TIAP, the patient complained of worsening dizziness. We discovered by X‐ray radiography that the catheter tip had entered the lower craniocerebral segment of the right IJV (Figure 3B). The patient's dizziness was relieved after the device was removed.

## 3 DISCUSSION

The use of TIAPs in both outpatients and inpatients has recently increased. Their main function is to deliver therapeutic drugs or parenteral nutrition solutions directly to the bloodstream so as to reduce the frequency of vascular puncture and irritation to blood vessels. Compared to traditional intravenous infusion through peripherally inserted central catheters, TIAP has fewer complications and lower costs and achieves greater patient satisfaction.^2^ Additionally, because the port is subcutaneously implanted, patients' privacy is protected and there is minimal disruption of their daily life activities.

The main puncture routes for TIAPs are the IJV, SV, and femoral vein. Although other locations have been reported, the right IJV appears safest.^3^ Catheter allotopia—typically immediate or postoperative spontaneous allotopia—is one of the most common complications of TIAPs, with an incidence of about 0.9%–1.8%. An analysis of 1619 cases of central venous catheterization reported an incidence of catheter allotopia of 3.3%, with catheter malpositioning most likely to involve the right SV (9.1%).^4^ One study analyzed 9346 patients who received TIAP from 2007 to 2016; of the 79 patients who underwent catheterization from the left side, the catheter tip strayed into AV in 7 (0.7%), while the incidence of left catheterization was 8.9%.^5^ In 2084 operations involving catheterization via SV, catheter malpositioning was the second most common early complication.^6^ A total of 350 TIAP insertions were performed in our inpatient department from January 2019 to June 2019, of which 25 cases (8.0%) involved left vascular access; the catheter migrated to AV in 1 case and to ITV in another, for a total incidence rate of 0.5%.

The 2 types of allotopia (immediate or postoperative spontaneous) differ in their causes. In addition to the implantation technique, severe cough, sneezing, tension, high flow rate of intravenous infusion, and catheter irrigation can lead to postoperative spontaneous allotopia, which may be related to an increased intrathoracic pressure.^7^, ^8^ The occurrence of immediate allotopia is closely related to vessel puncture and anatomic location. In one report, among 11 cases of catheter allotopia 4 were immediately ectopic and in the other 7, the catheter was displaced from the superior vena cava to AV for an average of 43 days.^9^ A jugular vein access port was associated with a significantly lower tip migration rate than SV.^10^ The AV insertions were most often in the posterior wall of the superior vena cava and occasionally in the right posterior wall. Therefore, when angioaccess is at an appropriate angle with respect to the left IJV or SV, the catheter can migrate into AV. Body mass index and age‐related changes in venous angle and posture have also been linked to catheter allotopia.^11^ Our previous studies showed that left side vein access is an important risk factor for catheter migration, with right vascular access—especially via the right IJV—having a lower incidence of catheter allotopia.^9^ There were a few cases in which the catheter tip migrated into ITV. Variations in vessel position are one possible cause of catheter allotopia; for example, Case 2 described in the present study had a thickened left ITV.

Potential complications associated with catheter allotopia include infection, venous perforation, local phlebitis, thrombosis, vascular stenosis or occlusion, vascular fistula, drug extravasation, and in rare instances, catheter rupture and cardiac allotopia. Dizziness as in our Case 3 is another often‐overlooked complication. The incidence of azygos vein perforation has been reported to be as high as 19%.^12^ Given the small diameter of the venous tube, there will be resistance when pushing the guidewire, and patients may experience chest tightness. Operators should be aware of these potential situations.

In view of the above complications, catheter allotopia should be prevented and managed in a timely manner. The best location for insertion of the catheter tip is at the junction of the superior vena cava and right atrium. Puncture based on body surface landmarks can lead to complications such as pneumothorax, hematoma, and nerve injury; therefore, echo‐guided vein puncture is currently accepted as the best method for decreasing major immediate complications during infusion port surgery. X‐ray radiography as well as DSA can be used to visualize the position of the catheter tip in real time and are thus important auxiliary tools.^13^ The success rate of radiologic and imaging‐guided implantations is >99%.^14^ In addition to these technologies, surface measurement, intracardiac electrocardiogram (IECG), and tracheal bifurcation methods are all reliable means of monitoring catheter tip position, with IECG being the most accurate.^15^ According to the literature and our experience, once catheter tip allotopia is detected after surgery, the first step is to remove the suture, partly retract and then reinsert the catheter, and then use X‐ray radiography to verify whether the position has been corrected. In cases where the position is not optimal, the lower segment of the catheter can be penetrated using a 21G needle that reaches the superior vena cava and then sends the catheter along the 0.018‐inch guidewire under fluoroscopic guidance. Importantly, the catheter tip position should be verified before each insertion to avoid postoperative spontaneous allotopia. Additionally, improving patients' knowledge of TIAPs can reduce anxiety and is as essential as postoperative port nursing.^16^

## 4 CONCLUSION

Totally implantable access ports are an efficient, safe, and economical tool for intravenous infusion. The occurrence of immediate allotopia is closely related to the choice of the puncture vessel, while postoperative spontaneous allotopia is related to incorrect use of TIAPs and increased intrathoracic pressure. Early detection and treatment are critical for preventing catheter allotopia and whenever possible, the right IJV should be selected as the puncture point. Intraoperative ECG monitoring and fluoroscopic guidance are strongly recommended when the TIAP is inserted from the right or left SV or IJV to ensure safe and error‐free positioning. If catheter malpositioning occurs during or after the operation, it must be immediately corrected and the catheter tip position should be verified by X‐ray or DSA radiography to avoid the risk of postoperative spontaneous allotopia.

## CONFLICT OF INTEREST

None declared.

## AUTHOR CONTRIBUTIONS

GJL and WGL: contributed to concept, design, procedures, and writing of the manuscript. HCH and HJG: contributed to procedures, resources, and writing of manuscript. LLC, JY, and YJL: contributed to concept, supervision, review, and editing of the manuscript.

## ETHICAL APPROVAL

The study was approved by the Institutional Review Board of Jiangsu Province Hospital on Integration of Chinese and Western Medicine. Informed consent has been obtained from all patients for the publication.

## Data Availability

Data sharing not applicable to this article as no datasets were generated or analyzed during the current study.
